# Predictive Distractor Processing Relies on Integrated Proactive and Reactive Attentional Mechanisms

**DOI:** 10.1523/JNEUROSCI.0740-25.2025

**Published:** 2026-01-19

**Authors:** Oscar Ferrante, Ole Jensen, Clayton Hickey

**Affiliations:** ^1^Centre for Human Brain Health, School of Psychology, University of Birmingham, Birmingham B15 2TT, United Kingdom; ^2^School of Psychology, University of Surrey, Guildford GU2 7XH, United Kingdom; ^3^Oxford Centre for Human Brain Activity, Department of Psychiatry, University of Oxford, Oxford OX3 7JX, United Kingdom; ^4^Department of Experimental Psychology, University of Oxford, Oxford OX2 6GG, United Kingdom

**Keywords:** distractor suppression, magnetoencephalography, MVPA, predictions, statistical learning, visual attention

## Abstract

Visual attention is shaped by statistical regularities in the environment, with spatially predictable distractors being proactively suppressed. The neural mechanisms underpinning this suppression remain poorly understood. In this study, we employed magnetoencephalography and multivariate classification analysis to investigate how predicted distractor locations are proactively processed in the human brain. Male and female human participants engaged in a statistical learning visual search task that required them to identify a target stimulus while ignoring a color-singleton distractor. Critically, the distractor appeared more frequently on one side of the visual field, creating an implicit spatial prediction. Our results revealed that distractor locations were encoded in temporo-occipital brain regions prior to the presentation of the search array, supporting the hypothesis that proactive suppression guides visual attention away from predictable distractors. The neural activity patterns corresponding to this presearch distractor processing extended to postsearch activity during late attentional stages (∼200 ms), suggesting an integrated suppressive mechanism. Notably, this generalization from pre- to postsearch phases was absent in the early sensory processing stages (∼100 ms), suggesting that postsearch distractor processing is not merely a continuation of sustained proactive processing but involves re-engagement of the same mechanism at distinct stages. These findings establish a mechanistic link between proactive and reactive processing of predictable distractors, demonstrating both shared and unique contributions to attentional selection.

## Significance Statement

In a world full of distractions, anticipating and ignoring irrelevant stimuli is crucial. The brain suppresses distractions both proactively (by preparing for expected distractions) and reactively (by responding after they appear). Yet, how these processes interact is unclear. In this study, we used magnetoencephalography and multivariate classification during a visual search task, where distractors appeared more frequently on one side, enabling unconscious learning of their likely location. Our results indicate that the brain encodes the distractor's location even before the search begins, showing proactive processing. Moreover, we found a connection between this early suppression and the brain's later response to the distractors, suggesting that proactive and reactive distractor processing rely on shared mechanisms.

## Introduction

We are constantly overwhelmed by distractions, from persistent smartphone notifications to frequent advertisements. These can interfere with high-intensity goal–directed behaviors, like driving a car or operating heavy machinery, making it critical to understand how the brain anticipates and minimizes their impact. One way the brain achieves this is through visual attention, which relies on distinct cognitive and neuronal mechanisms for the selection of targets and suppression of distractors (Chang and Egeth, 2019; Van Moorselaar and Slagter, 2020; Hickey et al., 2025). A particularly striking form of distractor suppression is driven by expectations formed from prior experience with distractors (Ferrante et al., 2018; Theeuwes et al., 2022). When salient distractors are constantly presented with higher probability at specific locations within a visual search array, the attentional priority of those locations is reduced, thereby decreasing interference from future distractors appearing there (Hickey et al., 2014; Ferrante et al., 2018, 2023a; Sauter et al., 2018; Wang and Theeuwes, 2018b). This form of learned suppression usually occurs without participant awareness of the distractor location manipulation, suggesting a form of statistical learning (Perruchet and Pacton, 2006; Frost et al., 2019). It likely reflects a dynamic adjustment of weights in topographic priority maps that guide spatial attention allocation (Ferrante et al., 2018, 2023a; Wang and Theeuwes, 2018a; Britton and Anderson, 2020; Liesefeld and Müller, 2021; Duncan et al., 2023).

Statistical learning of distractor suppression has been suggested to rely on proactive mechanisms that act before stimulus onset (Ferrante et al., 2018; Huang et al., 2022). For example, high-probability distractor locations have been associated with reduced amplitude of the distractor positivity (Pd; Van Moorselaar and Slagter, 2019), an event-related potential (ERP) component associated with reactive (i.e., in response to a stimulus) distractor suppression (Hickey et al., 2009). The logic here is that the observed reduction in reactive distractor suppression is made possible by prior application of proactive suppression. However, until recently, this proactive suppression was not directly identified in brain data, and, as a result, this interpretation has been open to debate (Wang et al., 2019).

In this context, we recently tested the hypothesis that statistical learning of distractor suppression involves proactive modulations in the visual cortex (Ferrante et al., 2023b). We applied a novel approach to magnetoencephalography (MEG) data analysis, Rapid Invisible Frequency Tagging (RIFT; Zhigalov et al., 2019; Seijdel et al., 2023), to probe neural excitability in the visual cortex. Our results revealed a reduced RIFT response in the early visual cortex contralateral to high-probability distractor locations. These findings suggest that statistical learning of distractor suppression is mediated, at least in part, by the proactive downregulation of responsivity in the early retinotopic visual cortex representing high-probability distractor locations. Thus, learned distractor suppression appears to modulate both proactive and reactive suppression mechanisms. However, our understanding of the relationship between these instances of suppression remains rudimentary. Specifically, it is unclear whether proactive and reactive suppression are supported by independent neuronal mechanisms or reflect the same underlying operation that emerges at different times.

Here, we employ multivariate classification of MEG data to test whether proactive and reactive distractor suppression leverage shared or distinct neural mechanisms. We first tested whether the location of predictable distractors could be classified from presearch brain activity on a trial-wise basis. If the same level of proactive suppression is applied regardless of where the distractor will appear, one would not expect above-chance classification of the distractor location in the presearch interval. In contrast, if proactive attentional mechanisms bias processing on a trial-wise basis, the predicted location of the upcoming distractor may be reflected in neural activity prior to distractor onset. We further characterized the relationship between proactive and reactive distractor processing by conducting a temporal generalization analysis. If proactive and reactive distractor suppression rely on similar attentional mechanisms, we would expect temporal generalization between presearch and postsearch time windows. Conversely, a lack of such generalization would suggest that proactive and reactive suppression are governed by independent mechanisms.

## Materials and Methods

### Participants

The current paper is based on a novel analysis of MEG data first reported in Ferrante et al. (2023b). Twenty healthy volunteers (13 females) were recruited from the University of Birmingham community. All participants provided informed consent and met local inclusion criteria for MEG studies. The study was approved by the University of Birmingham STEM Ethics Committee.

### Experimental design

**Figure 1.**
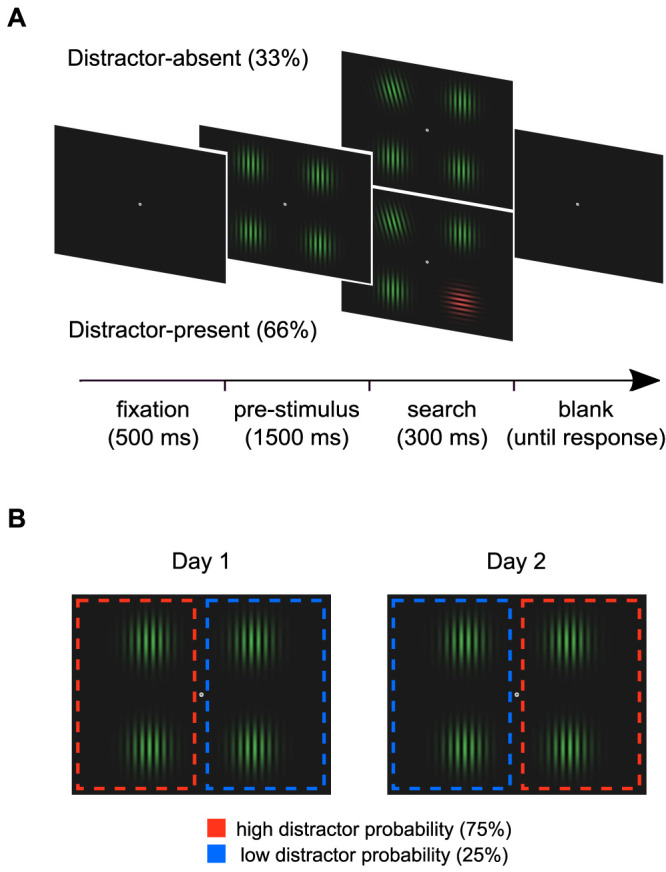
***A***, Visual search task. Each trial began with a fixation dot, followed by a placeholder display containing four identical Gabor patches. Afterward, a search display was presented and participants had to report the target orientation (i.e., left or right). In 66% of the trials, a color-singleton distractor appeared in the search display. ***B***, Statistical learning manipulation. The distractor appeared more often in one hemifield (75%) than the other (25%). Each participant completed two sessions of the experiment (Day 1 and Day 2), with the distractor probabilities swapped between hemifields in the second session (adapted from Ferrante et al., 2023b).

We used a variant of the additional-singleton visual search task (Theeuwes, 1992). Each trial started with a 500 ms fixation point, followed by a placeholder display presented for 1,500 ms, consisting of four identical nontarget stimuli positioned equidistantly at 4° of the visual angle (v.a.) from central fixation (Fig. 1*A*). The nontargets were vertically oriented Gabor patches of size 6 × 6° v.a. displayed in either red or green. After the offset of the placeholder display, a search display was presented for 300 ms. The target stimulus was identified as the Gabor patch tilted 15° to the left or to the right of the vertical meridian. In 66% of the trials, a horizontally oriented color-singleton distractor (e.g., a red distractor with green target and nontarget stimuli) was also presented. Participants had to identify the orientation of the target while ignoring the color-singleton distractor. A new trial started after an intertrial interval of between 500 and 1,500 ms (random, equal probability distribution).

To induce the statistical learning effect, a probabilistic bias was introduced to the location of the distractor stimuli (Fig. 1*B*). Specifically, we presented the distractor on the one side of the search display in 75% of trials and on the opposite side in 25% of trials, with the distractor equally likely to appear at one of the two possible positions within each hemifield. The target stimulus was presented equally across the two hemifields (50% each) and evenly distributed across the two possible locations within each hemifield. Each participant performed the task in two different sessions, with the distractor probability assignment swapped across sessions. To minimize carryover effects from the first to the second session, sessions were separated by 1–7 d.

As one goal of the experiment was to examine the RIFT signal, frequency tagging was implemented by flickering the two bottom stimuli at very high frequencies (>50 Hz) in the interval from the placeholder display until the offset of the search display. These high frequencies are above the flicker fusion threshold, making the tagging invisible, and further detail on this manipulation is available in Ferrante et al. (2023b). Since our current analyses focused on brain activity below 50 Hz, this manipulation is not relevant to the current study.

Participants received verbal instructions at the beginning of each session and completed a practice block consisting of 16 trials. The experiment consisted of six blocks, each comprising 120 trials, for a total of 720 trials per session. At the end of the second session, participants filled out a questionnaire to evaluate awareness of the statistical learning manipulation.

### MEG data acquisition and preprocessing

MEG data were acquired using a 306-sensor TRIUX system (MEGIN). MEG data acquisition was conducted following the FLUX Standard Operation Procedure (Ferrante et al., 2022). The data were sampled at 1,000 Hz with an online bandpass filter from 0.1 to 300 Hz. Four head position indicators (HPIs) were attached to the participant's head. The locations of the HPIs, fiducials, and the participant's head shape were digitized using a Polhemus Fastrack system (Polhemus). Eye movements were monitored using an EyeLink 1000 system (SR Research). Electrocardiography (ECG) and electrooculography (EOG) signals were recorded along with the MEG data. Manual responses were collected using two MEG-compatible response boxes (Nata Technologies).

MEG data were initially converted to BIDS (Niso et al., 2018) using the MNE-BIDS toolbox (Appelhoff et al., 2019) and preprocessed using the MNE-Python toolbox (v1.3.1; Gramfort, 2013) in accordance with the FLUX pipeline (Ferrante et al., 2022). Bad sensors were identified and reconstructed using a semiautomatic detection algorithm, after which signal-space separation (Taulu et al., 2003) was applied to reduce environmental noise. Independent component analysis was performed using the *fastica* algorithm (Hyvarinen, 1999). ECG and EOG were used to identify and project out independent components associated with cardiac and ocular artifacts, respectively. Finally, the MEG data were segmented into 4 s epochs, ranging from 1.5 s before to 2.5 s after the onset of the search display, with time 0 marking the onset of the placeholder display. Epochs with signal amplitudes exceeding an empirical peak-to-peak threshold obtained from the individual MEG data were excluded from the analysis. MEG data were then bandpass filtered between 1 and 50 Hz and resampled to 200 Hz prior to further analysis.

### MRI data acquisition and preprocessing

MRI anatomical images were acquired from each participant using a 3 Tesla Siemens Prisma scanner (T1-weighted MPRAGE; TR, 2,000 ms; TE, 2.01 ms; TI, 880 ms; flip angle, 8°; FOV, 256 × 256 × 208 mm; isotropic voxel, 1 mm). A single shell boundary element model (BEM) was constructed based on the brain surface derived using FreeSurfer (Dale et al., 1999). The BEM was then used to construct a volumetric forward model (5 mm grid) covering the full brain surface. The lead field matrix was then calculated according to the head position with respect to the MEG sensor array.

### Multivariate decoding analysis

We employed multivariate classification of MEG data to classify whether the distractor was presented in the left or right visual hemifield. We used a linear support vector machine (SVM) classifier from the scikit-learn toolbox (Pedregosa et al., 2011). For each time point, the distractor location was classified using data from all 306 MEG sensors within a 25 ms time window (five samples around each time point, resulting in a feature vector of 306 × 5 = 1,530 elements). Note that the use of this 25 ms sliding window, along with subsequent temporal clustering, can shift apparent effects slightly earlier than the actual stimulus onset; early decoding observed just prior to the search array should therefore be interpreted cautiously. To further enhance the signal-to-noise ratio, we created pseudotrials by averaging five randomly selected trials without replacement from each training and testing sets separately. To account for the higher number of trials in the high distractor probability condition, we subsampled these trials independently for each participant, randomly selecting a set of trials of the same size as that available in the low-probability distractor location. The resulting data were standardized as *z*-scores. Classification performance was quantified using the area under the curve (AUC) of the receiver operating characteristic, which evaluates the classifier ability to distinguish between the two classes (left vs right). A fivefold cross-validation approach was employed to ensure robust performance estimates, with the data randomly partitioned into folds. Classification was performed independently for each session, and the resulting values were then averaged across participants before statistical analysis. The same procedure was applied for classifying the target location.

To estimate the neuronal sources underpinning each classification activity, we repeated the classification analysis in source space (for a similar approach, see Lu et al., 2024). Source modeling was performed on individual MEG data using depth-weighted minimum norm estimates (MNE; Wang et al., 1992; Hämäläinen and Ilmoniemi, 1994) combined with dynamic statistical parametric mapping (dSPM; Dale et al., 2000). Epochs were baseline corrected using a 500 ms time window preceding the onset of the placeholder display. Noise covariance matrices were computed over this baseline time window, while covariance matrices for the presearch time window were computed using data from the onset of the placeholder display to 500 ms after. This was done by first estimating the rank of the data and then creating a common spatial filter by combining the baseline and presearch covariance matrices. MEG data were spatially prewhitened using the baseline covariance matrix, which allowed for the combination of gradiometer and magnetometer sensors (Engemann and Gramfort, 2015). The MNE–dSPM inverse operator was computed with a loose orientation of 0.2, a depth weighting of 0.8, and applied to the epoched data. The lambda2 regularization parameter was set to 1 (SNR = 1), and the source dipole magnitude was constrained to the normal orientation. Classification was then conducted on the source-level data using the same linear SVM decoder described above. To enhance the signal-to-noise ratio, each time point was classified within a 25 ms time window, data were averaged across every five randomly selected trials without replacement, and the top 500 features were selected through univariate feature selection (i.e., *F* test) and submitted to the classifier. The resulting source-level decoding weights were transformed into physiologically interpretable classification patterns (Haufe et al., 2014). Finally, individual source-level classification patterns were morphed to the FreeSurfer averaged brain (*fsaverage*) for group-level comparisons.

We additionally conducted multivariate temporal generalization analysis (King and Dehaene, 2014) to examine the temporal relationship between presearch and postsearch distractor location representations. The temporal generalization relied on multinomial logistic regression with fivefold cross-validation. The classifier was trained on data from a specific time point and then tested on all other time points across the 306 MEG sensors. Similarly to classification, this analysis was conducted within a 25 ms time window averaged over pseudotrials generated as mean averages of five randomly selected trials.

Cluster-based permutation tests (Maris and Oostenveld, 2007) were conducted to statistically assess above-chance classification performance. Two-tailed one–sample *t* tests were applied to each data point using a significance threshold corresponding to *α* = 0.05. Clusters were then formed by grouping adjacent significant data points. Cluster-level statistics were computed by summing the *t* values within each cluster. Statistical significance was calculated using a Monte Carlo null distribution calculated from 1,024 permutations of the data with randomization of conditional labels.

### Data and code accessibility

Raw MEG data are available on request. The experiment code is available at https://github.com/oscfer88/dSL_RIFT/tree/main/experimental_paradigm, and the code for performing all analyses can be found at https://github.com/oscfer88/predictive-attention-decoding.

## Results

### Proactive encoding of distractor location in presearch brain activity

We used temporally resolved classification to determine if the location of distractor stimuli could be identified in the MEG signal. The high-probability location is thought to be proactively suppressed: early visual neurons representing this position show reduced excitability (Ferrante et al., 2023b), and when a distractor ultimately appears here, it has less impact on behavior (Ferrante et al., 2018). We approached analysis with the expectation that this suppression might have a correlate in the pattern of neural activity observed in the presearch time window. Specifically, we asked whether the distractor location could be identified in classification analysis of MEG data before its appearance. Significant decoding would imply a predictive neuronal mechanism that allows attention to be proactively steered away from the anticipated distractor location. Conversely, chance-level decoding would indicate that the same suppression is applied to all locations.

**Figure 2.**
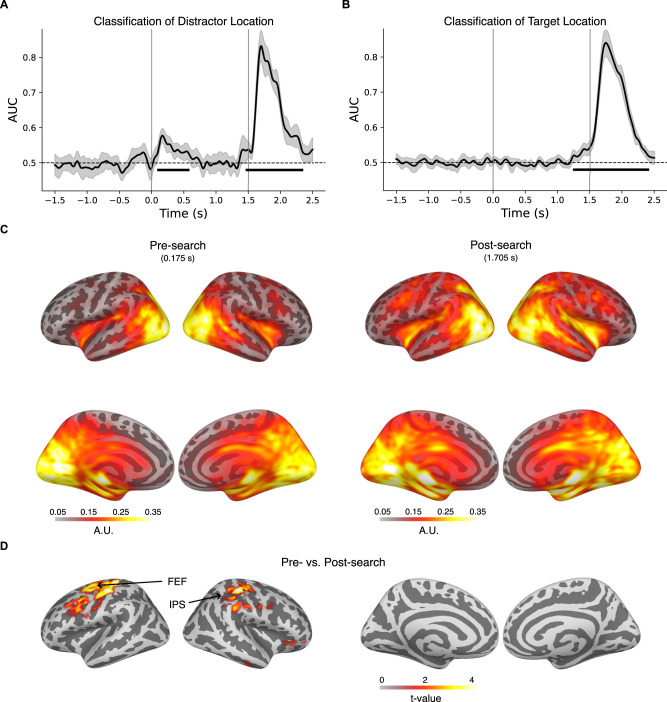
***A***, Time-resolved classification of distractor location in the current trial (left vs right hemifield; *t* = 0 s is onset of placeholder array; *t* = 1.5 s is onset of search array). ***B***, Time-resolved classification of target location in the current trial (left vs right hemifield). ***C***, Source-space projections of distractor location classification patterns at peak latency for the presearch period (175 ms) and postsearch period (1,705 ms). Color bars indicate the level of activation in arbitrary units. ***D***, Statistical contrast in source space between pre- and postsearch distractor location classification patterns, displaying only clusters showing significant effects. Color bars indicate the level of activation in arbitrary units and *t* values. FEF, frontal eye field; IPS, intraparietal sulcus.

Results showed significant classification of distractor location in the postsearch time window (peak classification time, 205 ms after search display onset; Fig. 2*A*), indicating distinct brain activity depending on whether the distractor was presented in the left or right hemifield. More importantly, we were able to successfully classify the distractor location during the presearch time window, with peak classification emerging 175 ms after placeholder display onset (1,325 ms before the search array appeared). This suggests that the statistical learning of distractor suppression influenced the proactive encoding of the distractor location, providing predictive information about its likely location in the upcoming search array.

The same classification analysis was performed on the target location. Since the target stimulus was presented with equal probability across hemifields, we did not anticipate any proactive representation of its location. In this sense, the classification analysis of the target location serves as a valuable control for the distractor location analysis. For the target location, the classification analysis revealed significant above-chance classification in the postsearch time window (Fig. 2*B*), mirroring the results of the distractor location analysis in the same time window. However, contrary to the distractor location analysis, the analysis of the target location did not yield significant above-chance classification in the presearch time window (Fig. 2*B*). This distinction was further validated through a cluster-based permutation test comparing the classification time series of the distractor and target locations. Specifically, the presearch classification for the distractor location was significantly different from that of the target location, with a significant cluster emerging between 95 and 615 ms after the onset of the placeholder display.

Finally, we localized the neuronal sources underlying presearch distractor processing by replicating the classification analysis on source-level data and computing the corresponding physiologically plausible classification patterns (Haufe et al., 2014). At the peak of sensor-level classification in the presearch interval (175 ms), source-level classification identified a bilaterally distributed network across temporo-occipital regions, including the extrastriate cortex, inferior parietal lobule, and middle temporal areas (Fig. 2*C*, left). We observed additional clusters in medial regions including the hippocampus and middle-posterior cingulate cortex; however, these findings should be interpreted cautiously due to the challenges of accurately localizing deep sources in MEG. At the peak of sensor-level classification in the postsearch interval (205 ms), source-level classification identified a similar temporo-occipital brain network, though with wider distribution, alongside clusters in the prefrontal and superior parietal cortex (Fig. 2*C*, right). A cluster-based permutation test comparing pre- and postsearch source-level classification patterns (Fig. 2*D*) revealed reliable differences in frontoparietal regions, including the frontal eye fields (FEF) and the intraparietal sulcus (IPS). Specifically, postsearch classification was associated with more bilateral activation of these regions, indicating greater involvement of prefrontal and superior parietal regions in reactive than proactive distractor processing.

### Presearch intertrials history effects in statistical learning

We further explored how presearch activity could predict the upcoming distractor location by focusing on intertrial dynamics. We approached the current data with the idea that intertrial history may build over repeated experience to instantiate effects associated with statistical learning of distractor suppression.

**Figure 3.**
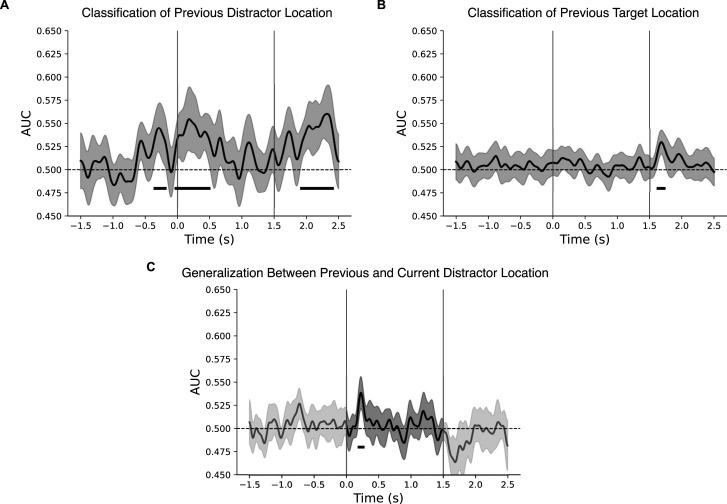
***A***, Time-resolved classification of the previous distractor location. ***B***, Time-resolved classification of the previous target location. ***C***, Time-resolved generalization between previous and current distractor location, obtained by training the classifier on the previous distractor location and testing it on the current distractor location. The gray line indicates classifier performance across the entire time window, while the black segment denotes the time period used for statistical testing (cluster-based permutation test). All classification results are presented as AUC with error bars depicting the 95% CI. Horizontal black lines indicate significant clusters. Vertical gray lines mark the onset of the presearch (0 s) and the search displays (1.5 s) and dashed black lines represent chance-level classification.

To test this hypothesis, we trained a sensor-level classifier to identify from data in the current trial (*N*) where the distractor appeared in the immediately preceding trial (*N*−1). Our goal was to determine whether information about the previous distractor location is reinstated in the presearch brain activity of the current trial. The results revealed three significant clusters of classification, spanning both presearch and postsearch time windows (Fig. 3*A*). This indicates that intertrial history plays a role in both proactive and reactive distractor processing. Notably, presearch classification was absent when the classification analysis focused on the previous target location (Fig. 3*B*), suggesting that the presearch intertrial effects were not a pure product of repeated experience but tied to the statistical regularity of distractor location. Examination of a 50 ms window ∼200 ms revealed a trend toward higher decoding of previous distractor versus target location (*t*_(19)_ = 2.08; *p* = 0.052; BF10 = 1.354). These results are in line with the hypothesis that statistical learning builds from intertrial dynamics.

Finally, we performed a generalization analysis to determine whether the above-chance classification of distractor location in the presearch interval reflected a partial reactivation of intertrial information. Specifically, we trained a classifier on current-trial MEG data to decode the location of the distractor from the previous trial (*N*−1) and tested it on the same data to decode the location of the distractor in the current trial (*N*). This approach allowed us to assess whether intertrial carryover, together with statistical learning, contributed to presearch decoding of upcoming distractor locations. Accordingly, the generalization analysis focused on the presearch interval (0–1.5 s). We observed significant generalization between previous and current distractor locations during this time window (Fig. 3*C*), with a cluster overlapping the main distractor location classification and peaking at 225 ms. These results indicate that proactive processing of the upcoming predicted distractor may arise from the combined influence of intertrial and statistical learning mechanisms.

### Proactive and reactive distractor processing generalize across attentional processing stages

**Figure 4.**
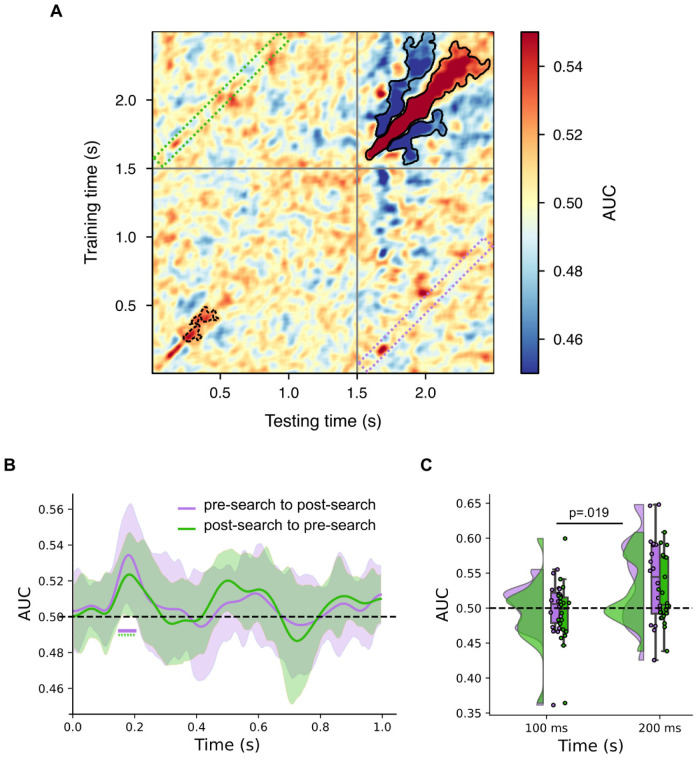
***A***, Temporal generalization of distractor location classification. Results are presented as AUC. Significant clusters (*p* < 0.05) computed over the entire epochs (0–2.5 s) are marked by solid borders. Significant clusters (*p* < 0.05) computed over the presearch time window (0–1.5 s) are marked by dotted borders. ***B***, Temporal generalization between presearch (0–1 s) and postsearch (1.5–2.5 s) time windows. The purple curve represents results obtained by training the decoder on the presearch time window and testing on the postsearch window (Fig. 3*A*, purple-dotted area). For this curve, graph origin reflects onset of a time interval beginning at onset of the placeholder display. The green curve represents results obtained by training the decoder on the postsearch window and testing on the presearch time window (Fig. 3*A*, green-dotted area). For this curve, graph origin reflects onset of a time interval beginning at the onset of the search display. Results are presented as AUC with error bars depicting 95% CI across subjects. Significant clusters (*p* < 0.05) are indicated by horizontal solid lines. Asterisks denote clusters with *p* < 0.10. Horizontal dashed line indicates the chance level. ***C***, Raincloud plot of temporal generalization centered at 100 and 200 ms delay for presearch to postsearch (purple) and postsearch to presearch (green). Each condition displays the full data distribution, individual datapoints, and the mean with error bars indicating the 95% confidence interval. Horizontal dashed line indicates the chance level.

We conducted a temporal generalization analysis to investigate whether the neural features supporting distractor location decoding in the presearch interval also contributed to its classification in the postsearch time window. The confusion matrix illustrating this analysis is presented as Figure 4*A*. Cluster-based permutation tests conducted on data over the entire time epoch revealed significant clusters in the postsearch time window that extended beyond the duration of stimulus presentation (300 ms), indicating distractor processing that continues after the offset of physical stimuli. A focused analysis of the presearch time window, from placeholder display onset (0 ms) to search display onset (1,500 ms), revealed comparable results during this period. These findings align with the primary classification results above, showing distinct clusters of classification accuracy in both pre- and postsearch intervals.

Temporal generalization emerges in the confusion matrix as off-diagonal clusters of classification accuracy. No significant off-diagonal clusters were observed (Fig. 4*A*), but this broad analysis has low statistical power due to the need for extensive multiple-comparison correction. As we approached the experimental data with a priori hypotheses regarding the relationship of presearch and postsearch distractor processing, we conducted additional focused analyses to test this idea. Here, we trained a classifier on sequential time windows in the interval following onset of the placeholder display and tested this classifier on corresponding time windows following onset of the search display and then did the opposite. This meant, for example, that the classifier trained to identify distractor location 200 ms after onset of the placeholder display was used to identify the distractor location 200 ms after onset of the search display and vice versa. This revealed temporal generalization between pre- and postsearch time windows when training on the presearch and testing on the postsearch time window, with a significant cluster emerging with a peak at 185 ms (Fig. 4*B*). A similar result was observed when we trained the classifier on the postsearch results and tested it on the presearch data, with a peak emerging at 175 ms, though this cluster did not independently reach statistical significance (cluster significance, *p* = 0.098).

We conducted a post hoc analysis on time windows traditionally associated with neuronal correlates of perceptual (e.g., P1; Clark et al., 1994; Hillyard and Anllo-Vento, 1998) and attentional processing (e.g., N2pc; Luck and Hillyard, 1994; Eimer and Kiss, 2007; Hickey et al., 2009). Specifically, we performed a series of one-sample *t* tests (FDR corrected) on the temporal generalization results, averaging the data over 50 ms time windows centered at 100 ms (i.e., 75–125 ms range) and 200 ms (175–225 ms range), for both directionalities (i.e., training on the pre- and testing on the postsearch time window and vice versa). This identified significant temporal generalization between the pre- and postsearch time windows in the 200 ms range (Fig. 4*C*; presearch to postsearch, *t*_(19)_ = 2.88; *p* = 0.019; postsearch to presearch: *t*_(19)_ = 2.21; *p* = 0.040). In contrast, temporal classification did not differ from chance in the 100 ms range for either analysis direction (presearch to postsearch, *t*_(19)_ = −0.17; *p* = 0.756; BF10 = 5.301; postsearch to presearch: *t*_(19)_ = −0.73; *p* = 0.763; BF10 = 1.671). The absence of generalization in this early window is unlikely to reflect reduced decoding strength alone, which depends on representational similarity rather than accuracy magnitude and may instead reflect differences in the underlying representational information, although firm conclusions are limited by the null result.

## Discussion

This study examines the neuronal mechanisms underlying the proactive processing of stimulus locations likely to contain distractors. Using MEG classification analysis, we find presearch activity in temporo-occipital regions reliably encodes upcoming distractor locations. This classification generalizes to postsearch processing in a time window associated with attentional selection (∼200 ms), suggesting that proactive and reactive distractor processing rely on shared attentional mechanisms.

Previous research has shown that distractor-specific statistical regularities influence the deployment of spatial attention (Ferrante et al., 2018; Theeuwes et al., 2022). These effects have been identified in overt behavior (Ferrante et al., 2018, 2023a; Sauter et al., 2018; Wang and Theeuwes, 2018a,b), in modulations of the distractor-elicited Pd ERP component (Van Moorselaar and Slagter, 2019; Wang et al., 2019), and in neuronal excitability (Zhang et al., 2022; Ferrante et al., 2023b; Richter et al., 2025). However, there are competing accounts of how distractors are ultimately suppressed (Luck et al., 2021). On the one hand is the idea that suppression avoids the capture of attention (Folk and Remington, 1998; Gaspelin et al., 2015; Gaspelin and Luck, 2018). On the other hand, is the notion that known distractors still draw attention but are quickly suppressed (Theeuwes, 2010; Moher and Egeth, 2012; Klink et al., 2023). Our findings here are compatible with both accounts: presearch classification could track proactive suppression but could also arise if expected distractor locations were transiently attended. However, this “search-and-destroy” account of attentional suppression is difficult to reconcile with our earlier results, where we found a reduction in presearch neural excitability in the cortex responsible for the representation of high-probability distractor locations (Ferrante et al., 2023b).

Our classification results reveal distractor suppression at two temporal intervals: (1) proactive suppression before search onset and (2) reactive suppression following distractor presentation (Luck et al., 2021; Liesefeld et al., 2024). This might suggest distinct physiological processes, with proactive suppression inhibiting early precognitive perceptual encoding and reactive suppression inhibiting semantic or task-related representations (Di Bello et al., 2022). However, our source modeling provides no evidence of this distinction. Both pre- and postsearch suppression were associated with similar patterns of brain activity, suggesting shared mechanistic basis. Temporal generalization analysis further reinforced this conclusion. Classifiers trained on presearch data successfully decoded distractor location from postsearch activity and vice versa. Cross-temporal classification emerged from ∼200 ms after the onset of either the placeholder or the search array—precisely the latency associated with attentional engagement (e.g., N2pc, Pd). Importantly, generalization did not emerge at earlier sensory latencies (∼100 ms), suggesting that suppression acts on attentional priority representations rather than low-level perceptual encoding. This is in line with recent results from analysis of steady-state potentials, which also shows evidence of distractor suppression from ∼200 ms (Duncan et al., 2025).

Motivated by this convergence, we propose that proactive, statistically learned suppression emerges from repeated reactive suppression and that this is shaped by intertrial dynamics. When a distractor appears at an unexpected location, reactive suppression leaves a transient inhibitory trace (Ipata et al., 2006; Cosman et al., 2018). Reappearance of the distractor at the same location produces a weaker neural response (Grill-Spector et al., 2006) and reduces the likelihood of attentional capture (Goschy et al., 2014; Turatto and Pascucci, 2016; Lega et al., 2020). When task performance is successful, reinforcement strengthens this trace (Hickey et al., 2014), allowing it to persist across trials. Over time, these repeated reactive adjustments accumulate into a stable proactive bias—a statistically learned suppression gradient encoded in the attentional priority map. Because this proactive, statistically learned suppression builds from reactive, intertrial suppression, the mechanisms share a neurophysiological profile.

By this account, we can classify upcoming distractor locations in the current results because the inhibitory trace from the previous trial is represented in the neural activity. Importantly, this is not a simple reflection of the repetition of stimulus location. If it were, we should be able to identify the location of the target in the preceding trial, and this is not the case. Statistical learning emerges only when the inhibitory tag is disproportionally reinforced by our manipulation of probability. Under these circumstances, it comes to sustain across trials.

This theoretic proposal identifies open questions. We have identified an interaction between intertrial dynamics and statistical probability that leads to statistical learning, but the precise relationship remains unclear. For example, we do not know how sensitive statistical learning is to continued repetition of intertrial contingencies. Presumably, when exceptions to the statistical probability occur and the distractor appears at a low-probability location, this leads to greater distraction in the subsequent trial. Is statistical learning simply reduced in this circumstance or is it entirely reset? We know that statistical learning of distractor location emerges quickly in behavior (Ferrante et al., 2018); how quickly do its neural effects extinguish?

Presearch classification of distractor location only emerges in our results after the placeholder display, not during the empty fixation interval. This suggests that proactive suppression may reside in a “silent state”—a subthreshold or latent representation that is reactivated by an external event (Stokes, 2015). The placeholder display may have acted as such a “ping,” reactivating the representation stored in neurons encoding spatial attentional priorities. This interpretation aligns with recent findings from Duncan et al. (2023), who used a manipulation of target location probability to induce statistically learned attentional priority (Geng and Behrmann, 2005; Jiang et al., 2013; Ferrante et al., 2018). The authors demonstrated that latent changes in attentional priority could be decoded from activity evoked by a “ping” presented during the intertrial interval. Our findings extend this finding by showing that regularities in distractor location can similarly alter the priority landscape, with this modulation becoming evident when the placeholder display “pings” the representation of distractor locations.

Our source-localization results localize presearch distractor suppression to a bilateral temporo-occipital network encompassing occipital, middle temporal, and inferior parietal regions that has been identified in earlier work (Hopfinger et al., 2001; Ruff and Driver, 2006; Seidl et al., 2012; Reeder et al., 2017; Lega et al., 2020). Ruff and Driver (2006) previously observed increased activity in the middle occipital gyrus in response to a visual cue signaling the location of an upcoming distractor. Recent intracranial EEG evidence from Lin et al. (2024) demonstrates rapid distractor-specific activity (∼220 ms postsearch) in superior and middle temporal gyri, amygdala, and anterior cingulate cortex, with weaker contributions from parietal and frontal cortices. Consistent with our findings, these temporal regions exhibited activity specific to distractor location representation, supporting the notion of a temporo-occipital network for proactive distractor processing. Statistical learning may therefore facilitate the suppression of predicted distractor stimuli by strengthening coupling within the ventral attentional network, a system specialized for detection of salient and unexpected stimuli (Corbetta and Shulman, 2002).

In contrast, postsearch distractor suppression engaged prefrontal and superior parietal regions, including the FEF and IPS. There are at least three possible mechanistic explanations for this difference. First, proactive suppression through statistical learning may not engage the strategic control mechanisms typically associated with FEF and IPS (Ipata et al., 2006; Suzuki and Gottlieb, 2013; Marini et al., 2016; Cosman et al., 2018; Lega et al., 2019). Instead, it may influence spatial attention through changes in priority maps localized in the posterior cortex (Li, 2002; Sprague and Serences, 2013). Second is the possibility that proactive suppression is strategic by nature but relies on a brain network that does not involve FEF and IPS. This is supported by recent reports of similar presearch classification during “willed” attention (Nadra et al., 2023, 2025). A final alternative is that the observed proactive effects may be mediated by regions other than FEF and IPS—like the superior colliculus, pulvinar (Krauzlis et al., 2013), and caudate nucleus (Hikosaka et al., 2000)—that do not elicit robust MEG signal. Clarifying the precise contributions of these regions will require future studies using higher-resolution recordings, such as intracranial EEG.

In conclusion, predictable distractor locations can be decoded from presearch neural activity, demonstrating that distractor suppression begins proactively. Temporal generalization shows that proactive and reactive suppressions reflect a common neural mechanism operating at different latencies. Statistical learning thus constructs predictive representations of distractor locations, supporting integrated proactive and reactive control of attention.
